# Prehospital Births in the Region of Southern Denmark—A Cohort From 2016 to 2024

**DOI:** 10.1111/aas.70351

**Published:** 2026-10-05

**Authors:** Louise Lind Pedersen, Helle Collatz Christensen, Jan Stener Jørgensen, Josefine Gradman, Signe Amalie Wolthers, Søren Mikkelsen

**Affiliations:** ^1^ Prehospital Research Unit, Prehospital South ‐ EMS, Region of Southern Denmark Odense Denmark; ^2^ Institute for Clinical Research, Faculty of Health Sciences, University of Southern Denmark Odense Denmark; ^3^ Prehospital Center Naestved Region Zealand Denmark; ^4^ Department of Clinical Medicine University of Copenhagen Copenhagen Denmark; ^5^ Department of Obstetrics and Fetal Medicine, Odense University Hospital University of Southern Denmark Odense Denmark; ^6^ Department of Womens' Health University Hospital of Southern Denmark Aabenraa Denmark; ^7^ Hans Christian Andersen Children's Hospital, Odense University Hospital Odense Denmark; ^8^ Department of Regional Health Research University of Southern Denmark Odense Denmark

**Keywords:** ambulances, emergency medical system, prehospital births

## Abstract

**Background:**

Unplanned births outside hospitals involve higher risks of complications for mothers and newborns and require special obstetric or pediatric skills. However, ambulance calls for childbirth are rare, making it difficult for ambulance staff to maintain their skills. In Europe, unplanned out‐of‐hospital deliveries constitute 0.10% to 0.61% of all births. The exact number of prehospital births in the Region of Southern Denmark remains unknown. The study aimed to determine the extent of unplanned prehospital births attended by the ambulance service outside hospitals in the Region of Southern Denmark from January 2016 through June 2024. We further aimed to identify where the unplanned pre‐hospital births took place and to report any birth‐related complications in the mothers or newborns encountered by ambulance staff.

**Methods:**

The study was a cross‐sectional study utilizing the pre‐hospital electronic Patient Medical Record system over 8 years in a mixed urban–rural area with a population of 1.2 million people. All prehospital medical records for ambulance missions dispatched for childbirths were manually examined. The study period spanned from January 2016 through June 2024.

**Results:**

Out of 1,137,222 ambulance dispatches, 3543 cases (0.31%) involved a dispatch code related to childbirth. Many labor incidents were considered uneventful by the prehospital personnel. Of all ambulance runs related to childbirth, we identified 310 births in the Region of Southern Denmark in which ambulance staff assisted with complicated out‐of‐hospital deliveries. An additional 10 labor cases were regarded as sufficiently complex that obstetric manoeuvres aimed at delaying the delivery of the baby were performed prehospitally, while the mother was rushed to hospital for delivery.

**Conclusion:**

Births outside the hospital while the mother is in the care of the EMS were rare, occurring in 0.03% of all ambulance missions. Most of the births took place without complications for both mother and neonate. In four cases, however, the neonate was in cardiac arrest and required resuscitation. Given the limited exposure to prehospital births, we suggest incorporating training in handling these rare events into routine training for prehospital caregivers.

**Editorial Comment:**

This cohort study presents obstetrical cases that have been managed first in the prehospital setting by ambulance personnel. Obstetrical outcomes related to this are presented, and there are considerations for what can be desirable as preparation and competencies for ambulance responses for these types of cases.

**Abbreviations**
Abbreviations: definition
CPAP: continuous positive airway pressure
CPR‐number: civil personal registration number
EMS: emergency medical services
EMT: emergency medical technician
ePMR: electronic patient medical record
HEMS: helicopter emergency medical service
MECU: mobile emergency care unit

## 1 Introduction

Unexpected birth outside a hospital or a planned home birth usually results in the request for an ambulance. These prehospital births are rare but are associated with an increased risk of complications for both mother and child [1, 2, 3, 4]. It can be difficult for ambulance personnel to maintain their skills in delivery management [3, 5, 6].

In Denmark, approximately 57,000 children are born each year [7]. A Danish pregnant woman can choose to give birth at three different locations: in a hospital ward (97% [8]), at a birth center or at home [9]. In Europe, there has been an increase in the proportion of births outside hospitals (i.e., planned and unplanned home births, births during transport, and births at private birth centers) in recent years [10, 11, 12, 13, 14]. European studies have estimated the prevalence of unplanned births outside hospitals to be between 0.19% and 0.68% [1, 15, 16]. In Denmark, the proportion of planned home births and planned births at private clinics increased until 2017, then remained relatively stable until 2021. During that period, due to COVID‐19, planned home births were discouraged [9, 17, 18, 19]. However, the proportion of unplanned births outside hospitals in Denmark remains unknown.

Unplanned births outside hospitals are rare but constitute a clinically significant event that can entail increased risks for both mother and child compared to in‐hospital births [3, 16, 20]. Infants born before arrival at the hospital have an increased risk of morbidity and mortality [1, 2, 16, 21, 22].

This cross‐sectional, population‐based study aimed to (1) determine the number of prehospital births and complicated labor courses attended by the ambulance service in the Region of Southern Denmark from January 2016 to June 2024. As well as (2) where the events occurred (in the ambulance, at home, etc.), (3) the presentation of the fetus (cephalic presentation, breech presentation, etc.), and (4) identifying the complications reported by ambulance staff in relation to the birth for both the child and the mother.

## 2 Methods

### 2.1 System Settings

In Denmark, approximately 6 million people [23] are distributed across five health regions. One of these regions is the Region of Southern Denmark, which has a population of approximately 1.2 million people [24] and is a mixed urban–rural catchment area.

The education to become an emergency medical technician (EMT) in Denmark typically takes 3 years and 7 months. The EMT student undertakes both theoretical instruction and practical placements at ambulance stations and across various hospitals [25, 26]. The education focuses, among other things, on the laboring woman and on the typical progression of childbirth. One of the practical placements is at an obstetric department [27]. After completing the training, EMTs in the Region of Southern Denmark attend a two‐day internal course on “Complicated pre‐hospital births and the acutely ill child.” Here, they will refresh their theoretical knowledge and practical skills related to potential childbirth situations and the management of an acutely ill child [27].

The Danish emergency healthcare system is publicly funded [28]. All urgent health‐related calls are handled by a healthcare professional, usually a nurse or a paramedic, at the emergency medical dispatch centers. Depending on the situation and severity, the appropriate prehospital response is determined within a three‐tiered system. This system consists of the basic prehospital resource, an ambulance and/or a rapid‐response vehicle staffed by a paramedic, a ground‐based mobile emergency care unit (MECU), or a helicopter‐based rapid‐response unit (HEMS) staffed by a specialist in anaesthesiology and a paramedic [28, 29]. In the Region of Southern Denmark, there are 39 ambulance stations [30] with 73 ambulances staffed by approximately 700 ambulance personnel and paramedics, as well as 6 MECUs staffed by approximately 120 anaesthesiologists. The four Danish HEMS supplement all five health regions. During and shortly after treatment, ambulance staff record an entry in the pre‐hospital electronic Patient Medical Record system (ePMR). These contain information on pre‐hospital findings, examinations, and treatments, as well as the hospital to which the patient is admitted [31]. The ePMR also includes the Danish Civil Personal Register number (CPR‐number), which serves as a unique patient identifier [32].

### 2.2 Design and Data Source

The study was a cross‐sectional, population‐based investigation of births or complicated labor courses that occurred while the mother was under the care of the ambulance service in the Region of Southern Denmark from January 2016 through June 2024. The births occurred either unexpectedly or so quickly that the laboring woman could not reach the maternity ward and therefore ended up giving birth at home or on the way to the hospital, or before a midwife arrived for a planned home birth. Additionally, all labor courses in which the birth of a child was postponed by specific obstetric manoeuvres performed by ambulance personnel were included.

### 2.3 Acquisition of Data

A preliminary dataset containing all missions related to childbirth was constructed by combining ePMR charts recording ambulance missions that met the dispatch criteria. Further, text mining was performed on all ePMR containing one or more of the following words registered in the free text fields.

The data set thus consisted of all ePMRs that fulfilled one or more of the criteria:

1. Ambulance missions related to childbirth (Coded under Danish Index Code A17.01–A17.09) [33].
2. One or more of the following terms are mentioned in one of the available free text fields in the ePMR:
  “birth” and/or “*born”
  “stillborn”
  “umbilical cord prolapse”
  “amniotic fluid leakage”
  “maternity nurse”
  “midwife”

or derivatives thereof.

Duplicate entries were excluded.

From this initial dataset, all relevant births and labor courses that occurred while the mother was under ambulance personnel's care were identified by manual review of the ePMR.

To distinguish between uncomplicated ambulance missions consisting of the pregnant woman being transported to the hospital before giving birth, missions in which a birth actually occurred, and complicated labor courses delaying the birth of a child, all prehospital medical charts were manually reviewed. The prehospital births were subsequently stratified into the following five categories:

- Birth of a child before ambulance personnel's arrival.
- Birth of a child with ambulance personnel and a midwife present.
- Birth of a child with ambulance personnel present but no midwife.
- Birth of a child where a midwife had called an ambulance due to maternal or infant complications.
- Complicated perinatal course requiring emergency intervention, for example, umbilical cord prolapse, but where the child was first delivered in the hospital.

Incidents in which a child was born in the presence of ambulance personnel were further examined through manual searches in the ePMR. Information about birth complications for the child (e.g., respiratory problems, umbilical cord around the neck, or neonatal resuscitation) and in the mother (e.g., postpartum haemorrhage) was collected. All variables were registered by manual review of the ePMR (see below).

### 2.4 Outcomes/Variables

The exposure was births that took place outside the hospital, either because the birth started unexpectedly or so quickly that the woman in labor could not get to the maternity ward and therefore ended up giving birth at home or on the way to the hospital, or before a midwife could arrive for a planned home birth. The births occurred while the ambulance staff were responsible for the treatment of the mother.

Primary outcomes were:

- To determine the number of births attended by the ambulance service outside the hospital in the Region of Southern Denmark from January 2016 to June 2024.
- To determine the number of labor courses in which specific obstetric manoeuvres were applied to delay the birth until hospital arrival in the Region of Southern Denmark from January 2016 to June 2024.

Secondary outcomes included:

- To assess where the pre‐hospital birth of a child or the obstetric relevant events took place (e.g., in the ambulance, at home, etc.)
- To evaluate the presentation of the fetus (cephalic presentation, breech presentation, etc.)
- To describe the prehospital complications and interventions reported by ambulance staff in relation to the birth for both the child (e.g., neonatal resuscitation, intubation, continuous positive airway pressure (CPAP), inotropic treatment, fluid infusions, and others) and the mother (e.g., haemorrhage).

Other variables

- Obstetric manoeuvres performed by healthcare personnel (e.g., management of shoulder dystocia during delivery or management of breech presentation) at the scene of the incident.
- Proportion of live births and neonates born without signs of life.
- Ambulance response time, time spent at the scene, and transport time.
- Intended home birth or unplanned home birth.
- Mother's parity.
- Gestational age of the neonate.
- Outcome of the prehospital intervention (birth, procedure abandonment, or rushing to the hospital).

### 2.5 Complications Defined

The occurrence of complications was defined as any birth with a presentation other than cephalic presentation; any obstetric manoeuvres necessary; prehospital complications and interventions in relation to the birth for both the child (e.g., neonatal resuscitation, intubation, continuous positive airway pressure (CPAP), inotropic treatment, fluid infusions, others) and the mother (e.g., haemorrhage); umbilical cord prolapse.

### 2.6 Statistical Analysis

Data were anonymised for statistical analysis.

Medians, quartiles, or ranges were reported.

The Chi‐square test was applied to assess differences between the categorical variables. A *p*‐value of < 0.05 was considered statistically significant.

StataBE 19.5 (StataCorp, College Station, Texas, USA) was used for all analyzes.

### 2.7 Ethics Statement

This study received approval from the Judicial Office of the Region of Southern Denmark (Ref. No. 24/40009). Approval to store data was obtained from the judicial office of Odense University Hospital (Ref. No. 24/47366). Under the Act on Processing of Personal Data, no consent is needed to use data already stored in the registry for register‐based studies approved by the Danish Patient Safety Authorities. Therefore, no additional approvals are required according to Danish law [34]. In addition to the necessary approvals, all data handling complied with Danish and European laws governing the processing of personally identifiable information [35, 36].

## 3 Results

Between January 1st, 2016, and June 30th, 2024, a total of 1,137,222 ambulance runs were recorded across the entire Region of Southern Denmark. In total, 9987 ePMR charts met one or more of the inclusion criteria. Manual review of these charts resulted in 3543 ambulance runs being related to childbirth (See Figure 1). In 18 cases, it was not possible to determine whether a birth had occurred from the ePMR; these cases were excluded. In total, a birth (before or after ambulance arrival) occurred in 822 incidents (0.072% of all ambulance runs).

**FIGURE 1:**
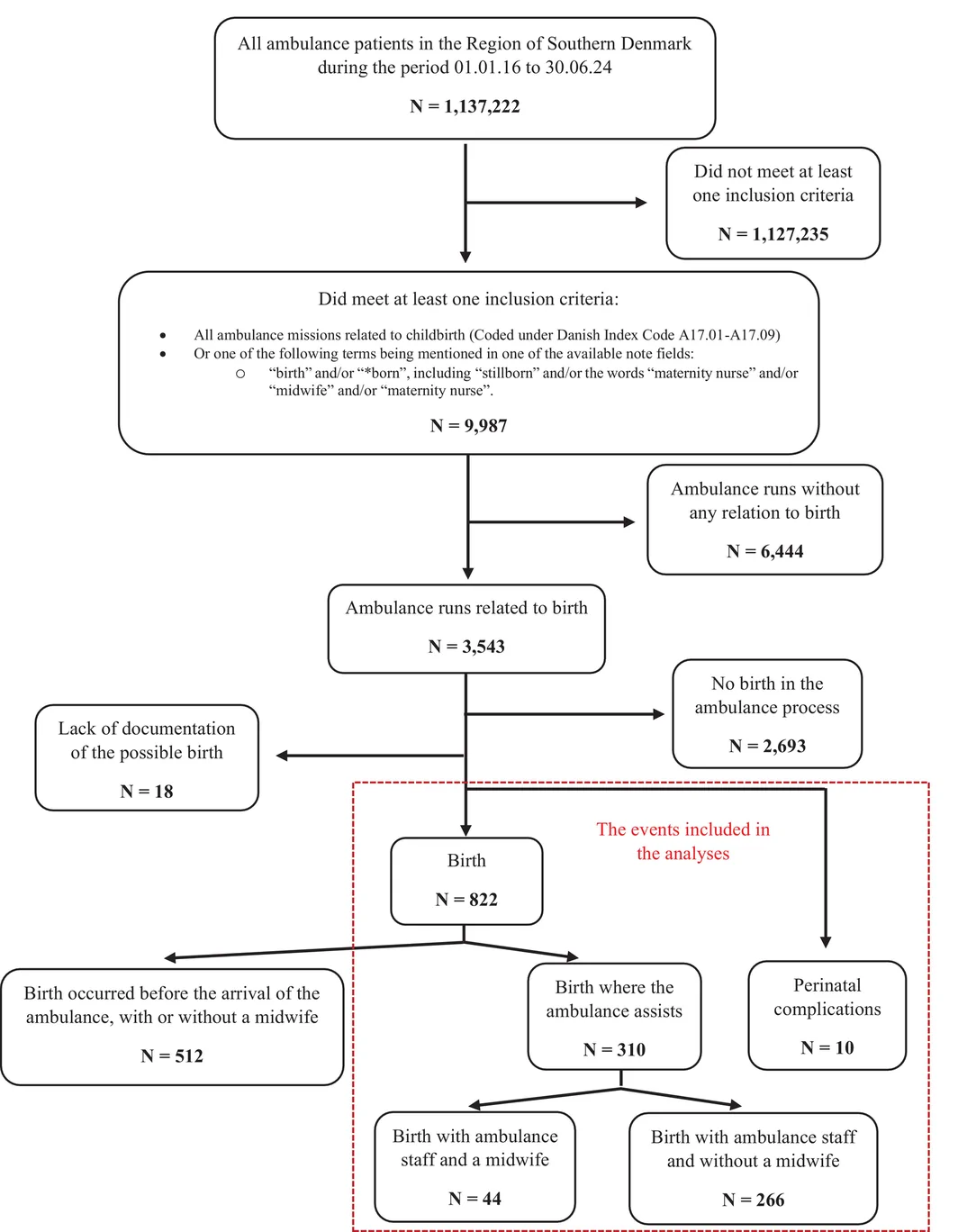
Flow chart of childbirths included in a study on prehospital births in the period of 2016 to 2024. The red dotted line delineates the events analyzed.

A total of 310 childbirths and 10 imminent births that required the mother to be rushed to the hospital during the ongoing delivery of a baby were relevant to the EMS.

### 3.1 Patient Demographic

The median age of mothers was 30 years (Quartiles: 27–33 years). In 8.8% of cases, the mother was giving birth for the first time. 45.9% of the women were giving birth for the second time, while 18.7% were giving birth for the third time or more (26.6% had unknown parity). The median gestational age was 40 weeks (Range 23–42 weeks). See Figure 2 for the distribution of gestational age at birth.

**FIGURE 2:**
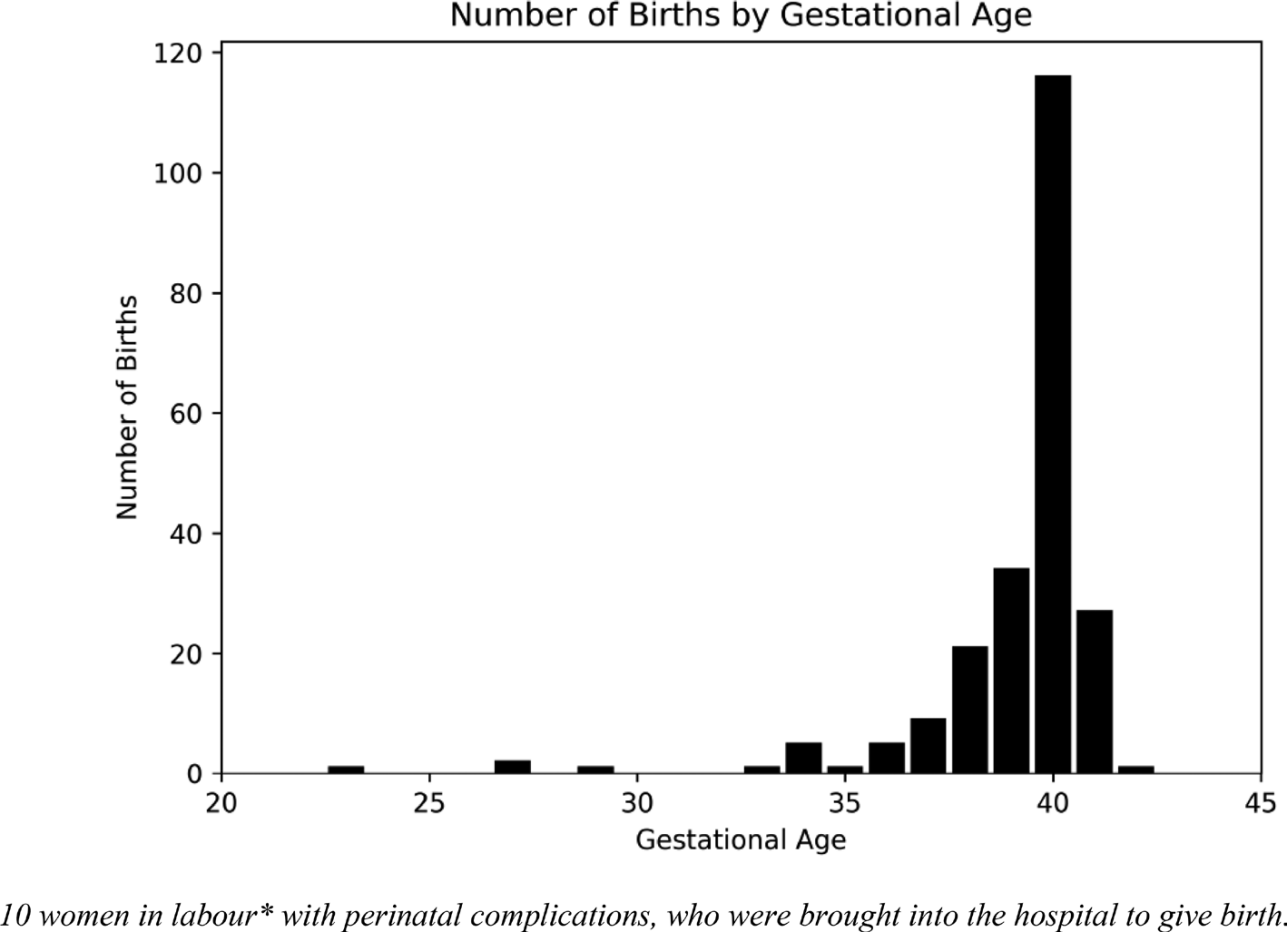
Diagram of the number of births by gestational age.

### 3.2 Time Spent at the Scene

The median response time was 7.5 min (Quartiles 5.4–11.0 min). Overall, the ambulance spent a median of 22.6 min at the scene (Quartiles 11.0–39.6 min). In cases where ambulance personnel attended to emergent severe on‐site perinatal complications and the woman was hurried to the hospital for the baby to be delivered at the hospital, the ambulance spent a median of 11.5 min at the scene (Quartiles 8.6–27.3 min). In cases where the neonate was delivered before transport to the hospital, the ambulance personnel spent a median of 22.7 min (Quartiles 11.1–40.6 min) at the scene.

### 3.3 Characteristics of the Children

In 286 (89.4%) births, the foetal presentation was cephalic. A minority of cases were breech, footling, and occiput posterior (star‐gazer) presentations. There were eight cases (2.5%) of prolapsed umbilical cord (see Table 1). Table 1 shows the distribution of birth locations.

**TABLE 1:** Presentation of the fetus and location where the birth took place.

Foetal presentation	*N*	Percent
Cephalic	286	89.4
Breech	5	1.6
Star gazer	1	0.3
Umbilical cord prolapse	8	2.5
Single footling presentation (no birth)	2	0.6
Unknown	18	5.6
Total	320	100.0

Place of birth	*N*	Percent
Home	182	56.9
In the ambulance	113	35.3
Car	7	2.2
Public place	1	0.3
In front of the hospital	4	1.3
At the hospital*	10	3.1
Unknown	3	0.9
Total	320	100.0

*Note:* Ten women in labour* with perinatal complications were brought into the hospital to give birth. The data include these 10 women with perinatal complications necessitating rapid transfer to a hospital.

277 (86.6%) of the births were planned at the hospital, but the neonate was born before arrival at the maternity ward. A small proportion of women, 28 (8.7%), had planned to have their birth outside the hospital, but due to the labor course progressing before the arrival of the midwife, an ambulance was called. In 15 births (4.7%), no information concerning planned or unplanned out‐of‐hospital birth could be derived. In 182 (56.9%) of cases, the births took place at home, and in 113 (35.3%) cases, in an ambulance (either just outside the house or the hospital, or during transport to the hospital). In the last 25 (7.8%) cases, the birth of the child occurred either in a car, a public place, just outside the hospital, within the hospital (the 10 cases that required the mother to be rushed to the hospital to deliver the baby because of complicated, imminent births), or the location was not documented in the ePMR.

In 241 (75.3%) cases, ambulance staff reported no complications in the neonate after birth. However, the five most common reported complications were: suctioning required in 29 neonates (9.1%), umbilical cord around the neck in 25 (7.8%), respiratory depression in 16 (5.0%), shoulder dystocia in eight (2.5%), and prolapsed umbilical cord without any birth in seven neonates (2.2%). Four children (1.3%) were born without any signs of life and required resuscitation. Six neonates had single footling presentation without birth (2), facial haematoma (1), umbilical cord rupture (1), umbilical cord prolapse with the mother giving birth prehospitally (1), and breech presentation with the fetus pushed back into the mother (1). In total, 95 complications occurred among 79 newborn babies.

### 3.4 Complications Regarding the Mother

In 254 incidents (79.4%), there were no critical complications requiring immediate treatment for the mother. The most common complication reported by the ambulance personnel was postpartum haemorrhage (19.7%), without other complications (11.3%) or with complications (8.4%). Some of the complications related to postpartum haemorrhage were shock, bimanual uterine compression, retained placenta following birth, or the mother required pharmacological treatment (e.g., tranexamic acid or oxytocin) to control the haemorrhage. All maternal complications are shown in Table 2.

**TABLE 2:** Critical perinatal complications observed prehospitally in the mothers.

Complication	*N*	Percent
None	254	79.4
Postpartum haemorrhage (without any other complications)	36	11.3
Postpartum haemorrhage with complications	27	8.4
Partial placental abruption	2	0.6
Prenatal haemorrhage	2	0.6
Perineal tear	1	0.3
Total	320	100.0

### 3.5 Midwife

In 254 incidents (79.4%), the entire birth took place without a midwife present. In 5.3% (*n* = 17) of the births, a midwife arrived after the delivery of the neonate, but before the placenta was delivered, and in 1.6% (*n* = 5) of the births, a midwife arrived after the delivery of both the neonate and the placenta. In 13.1% (*n* = 42) of the births, the ambulance personnel and the midwife delivered the neonate together. In 2 (0.6%) cases, incidentally, a midwife was present during birth (a neighbor or paramedic who had previously been trained as a midwife).

### 3.6 The Temporal Development in the Number of Prehospital Births Attended by Ambulance

We found no significant year‐to‐year variation in the number of births that required the presence of an ambulance during the years January 2016 to June 2024 (*p* = 0.14) (data not shown).

## 4 Discussion

### 4.1 Summary of Main Findings

Of all ambulance missions, 0.31% were related to childbirth. A birth (before or after ambulance arrival) occurred in 0.07% of all ambulance missions in the period. In slightly less than half of these missions (0.03% of all ambulance missions), the child was born after the arrival of the ambulance. Most of the women giving unplanned outside‐of‐hospital births were multiparous with a median age of 30 years; this is comparable to that of other Danish women giving birth [8]. The risk of prehospital birth was relatively low for first‐time mothers. The majority (86.6%) of births attended by the ambulance personnel were unplanned out‐of‐hospital births that took place in the woman's own home, without any complications for either the neonate or the mother.

### 4.2 Comparison With Other Studies

Births that occur outside hospital settings account for a small proportion of all ambulance calls. An Australian study reported that 0.5% ambulance missions were for pregnant women in labor. One in 10 of these missions resulted in prehospital childbirth. Among these ambulance missions, the birth occurred before the ambulance arrived in 42.4% of cases and during the journey in 57.6% [37]. In our cross‐sectional study, there were 3543 ambulance calls related to childbirth. Of these, 822 resulted in childbirth, equating to one birth in four birth‐related ambulance missions.

The geographical distance between the pregnant woman and the hospital offering obstetric services is the most significant risk factor for giving birth outside a hospital [1, 12, 38]. In the Region of Southern Denmark, there are five hospitals with maternity wards (see Figure 3). Comparing our findings in the Region of Southern Denmark with Australian findings, we found a lower proportion of ambulance runs related to births but a higher rate of actual childbirths. One may speculate that a shorter distance from a mother's home to the maternity ward could influence the timing of calls to emergency medical dispatch centers. Indeed, in other areas within Denmark, specifically the rural territory of Greenland, centralization of birthing mothers occurs as early as 3 weeks before the estimated birth [39].

**FIGURE 3:**
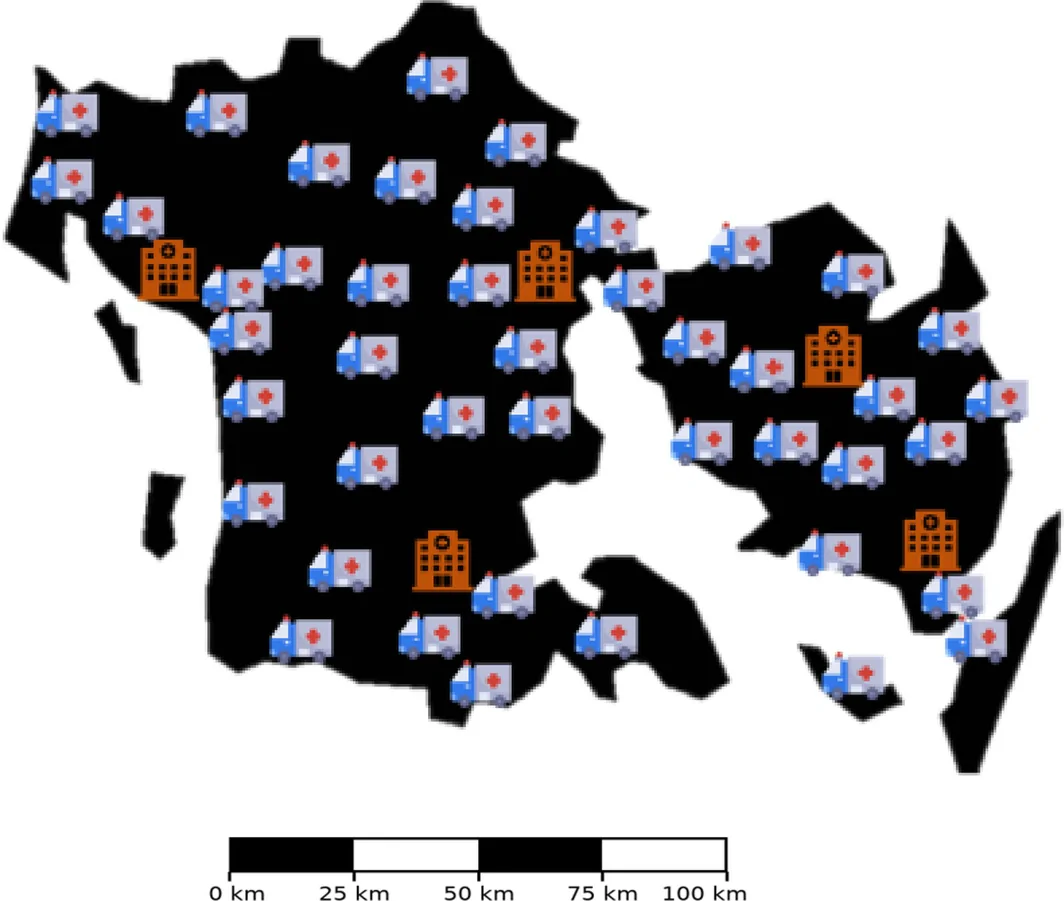
Map of the locations of maternity wards and ambulance stations in the Region of Southern Denmark. The inclusion of a scale bar provides an indication of the distances between maternity wards.

Predictors of neonatal morbidity and mortality have been reported to include multiparity, prematurity, maternal pathology, and hypothermia [38]. Overall, 64.6% of the women giving birth in our study were multiparous. The median duration of the first stage of labor in nulliparous women is twice as long as for multiparous women [40]. This may result in far more nulliparous women having time to reach the hospital before the birth of the child. Of the 320 children born prehospitally, 26 (8.1%) were born premature (before 37 weeks of pregnancy). Among these, four (1.3%) were extremely preterm, that is, born before 28 weeks of gestation.

### 4.3 Limitations and Strengths of the Study

The main limitation of this cross‐sectional study is that documentation in the pre‐hospital electronic Patient Medical Record system (ePMR) is mostly retrospective, due to the often urgent circumstances surrounding a nearby birth [41, 42]. This later documentation may result in recall bias.

A further limitation is that the search strategy was based on the data quality of the ePMR. Insufficient data in the ePMR would exclude the patient from the study.

Finally, the study includes no medical records for prehospital births involving HEMS because HEMS did not consistently use the same ePMR as the rest of the national ambulance services. This may result in some out‐of‐hospital births not being represented in this study.

A major strength of the study is that all medical records that met at least one inclusion criterion (*n* = 9987) were manually reviewed to determine whether a birth had occurred and whether the ambulance was directly involved in the delivery.

## 5 Conclusion

The average prehospital caregiver will only be exposed to few births. Given the potentially huge consequences of a perinatal event going wrong, it should be evident that routine cannot be maintained at the job. In line with previous studies on prehospital births [3, 5, 6], we suggest that training in handling these rare events should be incorporated into the routine training for prehospital caregivers to ensure that they possess adequate skills to manage situations where births do not proceed as expected (e.g., shoulder dystocia, breech presentation, postpartum bleeding, etc.).

## Author Contributions

**Louise Lind Pedersen:** conceptualisation, data collection, data analysis and interpretation, writing the first draft, critical revision of the draft, approval of the final manuscript. **Helle Collatz Christensen:** data analysis and interpretation, critical revision of the draft, approval of the final manuscript. **Jan Stener Jørgensen:** conceptualisation, data analysis and interpretation, critical revision of the draft, approval of the final manuscript. **Josefine Gradman:** conceptualisation, data analysis and interpretation, critical revision of the draft, approval of the final manuscript. **Signe Amalie Wolthers:** critical revision of the draft, approval of the final manuscript. **Søren Mikkelsen:** conceptualisation, data analysis and interpretation, critical revision of the draft, approval of the final manuscript.

## Funding

The study has received a Student grant from Odense University Hospital to cover half a year's salary for Louise Lind Pedersen, amounting to DKK 60,000. The Odense University Hospital Senior Medical Consultants' Fund and the Dr. Else Poulsen Memorial Scholarship have allocated DKK 25,000 for conference and publication expenses.

## Ethics Statement

This study received approval from the Judicial Office of the Region of Southern Denmark (Ref. No. 24/40009). Approval to store data was obtained from the judicial office of Odense University Hospital (Ref. No. 24/47366). Under the Act on Processing of Personal Data, no consent is needed to use data already stored in the registry for register‐based studies approved by the Danish Patient Safety Authorities. Therefore, no additional approvals are required according to Danish law. Besides the necessary approvals, all data handling complied with Danish and European laws regarding personally identifiable information.

## Consent

According to Danish legislation, no consent is needed to use data already stored in the registry for register‐based studies approved by the Danish Patient Safety Authorities.

## Conflicts of Interest

The authors declare no conflicts of interest.

## Data Availability

Data are available from the authors upon reasonable request within the limits of Danish legislation.
